# Functional electrographic flow patterns in patients with persistent atrial fibrillation predict outcome of catheter ablation

**DOI:** 10.1111/jce.15115

**Published:** 2021-06-07

**Authors:** Tamas Szili‐Torok, Zsuzsanna Kis, Rohit Bhagwandien, Sip Wijchers, Sing‐Chien Yap, Mark Hoogendijk, Nadege Dumas, Philip Haeusser, Tamas Geczy, Melissa H. Kong, Peter Ruppersberg

**Affiliations:** ^1^ Department of Cardiology Erasmus MC Rotterdam The Netherlands; ^2^ Ablacon, Inc. Wheat Ridge Colorado USA

**Keywords:** catheter ablation outcome, electrographic flow, persistent atrial fibrillation

## Abstract

**Aims:**

Electrographic flow (EGF) mapping is a method to detect action potential sources within the atria. In a double‐blinded retrospective study we evaluated whether sources detected by EGF are related to procedural outcome.

**Methods:**

EGF maps were retrospectively generated using the Ablamap® software from unipolar data recorded with a 64‐pole basket catheter from patients who previously underwent focal impulse and rotor modulation‐guided ablation. We analyzed patient outcomes based on source activity (SAC) and variability. Freedom from atrial fibrillation (AF) was defined as no recurrence of AF, atypical flutter or atrial tachycardia at the follow‐up visits.

**Results:**

EGF maps were from 123 atria in 64 patients with persistent or long‐standing persistent AF. Procedural outcome correlation with SAC peaked at >26%. S‐type EGF signature (source‐dependent AF) is characterized by stable sources with SAC > 26% and C‐type (source‐independent AF) is characterized by sources with SAC ≤ 26%. Cases with AF recurrence at 3‐, 6‐, or 12‐month follow‐up showed a median final SAC 34%; while AF‐free patients had sources with significantly lower median final SAC 21% (*p* = .0006). Patients with final SAC and Variability above both thresholds had 94% recurrence, while recurrence was only 36% for patients with leading source SAC and variability below threshold (*p* = .0001). S‐type EGF signature post‐ablation was associated with an AF recurrence rate 88.5% versus 38.1% with C‐type EGF signature.

**Conclusions:**

EGF mapping enables the visualization of active AF sources. Sources with SAC > 26% appear relevant and their presence post‐ablation correlates with high rates of AF recurrence.

**Abbreviations**
AF: atrial fibrillation
AT: atrial tachycardia
CA: catheter ablation
EGF: electrographic flow
FIRM mapping: focal impulse and rotor modulation mapping
ICE: intracardiac echocardiography
LA: left atrium
MEC: medical ethics committee
PVI: pulmonary vein isolation
PVs: pulmonary veins
RA: right atria
RAP: rotational activity profile
SR: sinus rhythm

## 1 INTRODUCTION

Consensus exists among electrophysiologists that performing pulmonary vein isolation (PVI) for patients with paroxysmal atrial fibrillation (AF) provides a highly effective treatment option.^1^, ^2^, ^3^, ^4^, ^5^ This procedure results in high acute and midterm clinical success rates and reasonably acceptable long‐term clinical outcomes.^1^, ^2^ However, the treatment of persistent AF remains more challenging. While catheter ablation reaches an almost 80% success rate at 12 months for paroxysmal AF patients in many recent trials, regardless of ablation strategy or ablation energy source, the long‐term success rates after ablation for persistent AF do not exceed 50%–55%.^6^, ^7^, ^8^, ^9^, ^10^, ^11^, ^12^ This may be related to incomplete understanding of the underlying atrial substrate outside the pulmonary veins (PVs). Another possible explanation is that existing mapping technologies cannot reliably and accurately detect active versus passive and/or sources of significance in this patient population.^13^, ^14^

Electrographic flow (EGF) mapping has been developed as a method to detect action potential sources in the atria of patients with AF.^15^, ^16^, ^17^ This novel technology has the potential to distinguish between active sources and passive rotational activities. Beyond this potential advantage of using this method, source statistics can also be analyzed during longer periods of AF to determine which active sources are actually significant and contributing to the perpetuation of fibrillatory activation in the atria.

The objective of this study was to use EGF mapping to identify different subtypes of persistent AF based on characteristics of the AF sources and to predict post‐ablation outcomes at 12 months based on the AF source subtype and whether or not relevant sources remained at the conclusion of the ablation procedure. As we are post‐processing multi‐electrode basket catheter recordings to create novel EGF maps, the goal is not to assess the effectiveness of focal impulse and rotor modulation (FIRM)‐guided ablation, but simply to characterize the sources present at baseline and the residual sources remaining at the end of the procedure that was performed. We hypothesized that the presence of active sources remaining at the end of a procedure means that extra‐pulmonary vein drivers of a patient's AF have not been eliminated and thus will likely result in AF recurrence; whereas the absence of any active sources remaining at the end of a procedure means that provided that PVI is durable, the patient should experience freedom from AF.

## 2 METHODS

### 2.1 Study design

This study is a double‐blinded retrospective study designed to use EGF mapping technology to visualize active sources remaining after an ablation procedure. All included patients previously underwent FIRM‐guided catheter ablation and had 12 months post‐ablation follow‐up data available. The raw intracardiac electrograms obtained from 64‐pole basket recordings were analyzed post‐hoc using the Ablamap® software (Ablacon Inc.) for EGF mapping, which was not available at the time of the original procedures. All electrocardiogram data files were anonymized, and both the analysts and the software operators were blinded to the available outcomes data. The local medical ethics committee (MEC) approved the data collection and concluded that the study did not fall under the Medical Research Involving Human Subjects Act.

### 2.2 Study population

#### 2.2.1 Ablation procedure

For the purpose of this study, we created a retrospective cohort of 64 patients who underwent FIRMmapping and ablation from March 4, 2015 through November 1, 2017 at Erasmus Medical Center in Rotterdam, the Netherlands. Although all of the cases included in this study were analyzed retrospectively, a brief summary of the ablation procedure workflow follows to contextualize the acute and long‐term outcomes of clinical ablation. Antiarrhythmic drug therapy was uninterrupted. For all patients, a decapolar catheter was advanced into the coronary sinus and if the patient arrived in the electrophysiology lab in sinus rhythm (SR), AF was induced with decremental atrial burst pacing. Intravenous heparin was administered to reach activated clotting time (ACT) > 300 sec before introduction of the basket catheter. Sustained AF of more than 5‐min duration was then recorded using a 64‐pole basket catheter (FIRMap™; Abbott) that was passed through an 8.5F SL1 sheath, first in the right atrium (RA) and then in the left atrium (LA). Sizing and positioning of the basket catheter, including the confirmation of acceptable atrial contact, were ensured by intracardiac echocardiography. If the signal quality was poor, the basket catheter was repositioned until adequate raw electrogram signals could be recorded. In all patients, first RA basket recordings and subsequent ablation as indicated by FIRM mapping followed by LA basket recordings and subsequent ablation as indicated by FIRM mapping was performed. The order of 64‐pole basket recordings and FIRM‐guided ablation and PVI was based on operator discretion. In the LA, 64‐pole basket recordings were repeated after PVI. The details of FIRM mapping are not relevant to this study as we are analyzing the raw electrograms recorded from the basket and 12‐month clinical outcome post‐ablation.

Using 3.5‐mm irrigated‐tip catheters, radiofrequency energy was applied based on the basket grid coordinates referenced to electrode positions on the three‐dimensional electroanatomic maps. The power setting varied between 25 and 40 W (posterior vs. anterior wall, respectively) and the temperature limit was set to 43°C. Verification of PVI was performed in all patients using a circular mapping catheter to confirm both entrance and exit block if the patient was in SR (Lasso™; Biosense Webster) after conventional PVI. If AF organized into atrial flutter or atrial tachycardia, then these were mapped and ablated in the standard fashion. Additional substrate ablation (roof or mitral isthmus line, non‐PV sources) was not performed. Electrical cardioversion was performed only in the absence of conversion to SR after completion of the ablation protocol.

#### 2.2.2 EGF mapping

EGF mapping is a novel method of visualizing cardiac action potential flow and the details of this method have previously been published.^15^, ^16^, ^17^ The ability to visualize action potential flow enables the identification and characterization of functional mechanisms of AF such as active sources that serve as drivers and/or triggers of AF. In many persistent AF patients, spontaneously active sources with focal or reentrant mechanisms repeatedly originate electrical excitations at defined locations in the atrial wall, which flow into the otherwise chaotic excitation waves of fibrillation. EGF mapping identifies these AF sources by detecting excitation waves traveling centrifugally from the center of the source in the atrial wall. EGF mapping can discern and visualize these regular centrifugal patterns from the chaotic excitation waves characteristic of AF by using a patented algorithm derived from computer vision optical flow analysis.

#### 2.2.3 Electrogram analysis and post‐processing

**Figure 1:**
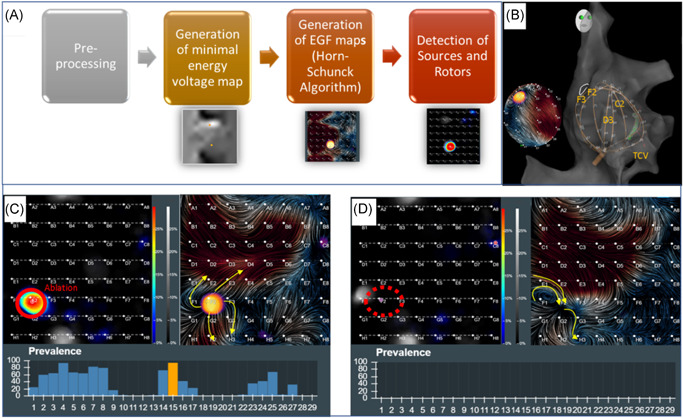
(A) Sequence of the processing to yield electrographic flow (EGF) maps. (B) Localization of atrial fibrillation (AF) source using basket catheter. (C) AF source visualization with prevalence map (left panel) and EGF map (right panel). (D) After successful ablation of the AF source visualized in (B) and (C), repeat EGF mapping post‐ablation shows that the active source is no longer present

EGF maps were created using the previously stored 1‐min recordings obtained with the above‐described 64‐pole basket mapping catheter according to a four‐step processing pipeline (Figure 1A) that takes no more than a couple seconds to complete. Unipolar and bipolar intracardiac signals from the basket catheter were filtered at 0.05–500 Hz and recorded at 1 kHz sampling frequency for export from the electrophysiology recording system. As described previously, the Ablamap software pre‐processes unipolar electrograms to remove far‐field artifacts and normalizes the signals to unitary amplitudes before they undergo flow analysis.^15^, ^16^ For flow analysis, each 19 ms of far‐field corrected and normalized recordings are summarized in a single minimal energy voltage map using Green's algorithm assuming micro‐electroneutrality and undisturbed spreading of electrical fields. Subsequent minimal energy voltage maps are then analyzed using the Horn–Schunck algorithm to determine the most likely EGF pattern. Representative EGF maps are determined for each 2‐s interval. 3045 EGF maps representing a full minute are analyzed with respect to flow patterns to yield an active source prevalence map over one full minute. The source with the highest prevalence is determined as the leading source.

#### 2.2.4 Definition of activity and variability of leading EGF sources

Source activity (SAC) is determined by counting the percentage of time during which a leading source was detected as active with its characteristic detection rate and being located within the range of one electrode distance during the minute of recording. Variability of the leading source is determined as the percentage of surface of the total recording area in electrode distance units necessary to cover 80% of the SAC. The potential improvement in mapping resolution as a result of the more closely spaced electrodes near the basket catheter pole is not significant because EGF‐based SAC is a time‐dependent and not a space‐dependent parameter.

Figure 1C shows a typical prevalence map (left) and an EGF map (right) displaying an active source (centrifugal flow; arrows indicate direction of flow lines) at electrode F2 from a recording made in the central RA with basket as shown in the CARTO (Biosense Webster) electroanatomical map in Figure 1B. The activity of the source as shown in the histogram bar plot changed over the course of the 1 min of recording with an average SAC of 35.4% and a variability of 2.8% signifying a singular, dominant, active, leading source. Detailed review of the operative report showed that the location F2 was ablated with 300 sec of RF ablation after which, another 1‐min basket recording was made in the same location. Post‐ablation, with unchanged position of the basket catheter, the prevalence map no longer displayed any activity in the previous source location (Figure 1D); however, the EGF pattern was very similar around the now passive area at F2 (centripetal inward flow). Post‐ablation, maximum SAC decreased to 10.3% and variability increased to 5%.

#### 2.2.5 Analysis of median AF cycle length (CL) pre‐ and post‐ablation of sources

Data from all channels of a basket recording were first filtered with 5 Hz high pass and 40 Hz low pass. All channels with an amplitude <0.8 mV were removed. QRS duration was detected and 200 ms around the steepest R slope was subtracted. Locations of f wave activations were identified by the maximal values of the signal first derivative and a histogram of the time intervals between successive maximal values was constructed. This histogram was then fitted with three Gaussians for each channel taken into account and the largest Gaussian amplitude from each channel of a recording was then used to calculate the median. For all sources with SAC above threshold, median AF CL pre‐ and post‐ablation was calculated and correlated with outcome.

#### 2.2.6 Follow‐up

Patients were seen in the outpatient clinic following a 3‐month blanking period at 3, 6, and 12 months post‐ablation. During these visits, 12‐lead electrocardiograms (ECGs) were obtained. In addition, long‐term monitoring was obtained by transtelephonic ECG monitoring between 3 and 4, and between 6 and 7 months post‐ablation. At 6‐ and 12‐month follow‐up, 7‐day Holter recordings were obtained. Between the 6 and 12 months of follow‐up, symptom‐driven event monitoring was performed as clinically indicated. Freedom from AF was defined as absence of AF or any other atrial arrhythmia other than cavotricuspid isthmus‐dependent RA flutter, lasting for >30 sec on one of the recordings described above.

#### 2.2.7 Statistical analysis

Statistical analysis was performed using Microsoft Excel software. Outcome dependence of SAC and variability was determined by first sorting all patients according to the respective parameter. Next, the percentage of accurately judged cases was plotted against the cut‐off threshold (above vs. equal or below) value and the result was fitted with a polynomial of the fifth order using Excel. The peak of the polynomial fits was defined as threshold value. Next, a *χ* ^2^ test was used to measure the *p* value for the hypothesis that there is a difference in outcome of cases above and below, respectively, equal to the threshold. Statistical difference of recurrent and AF‐free cases with respect to the parameters was tested using single‐sided Student's *t*‐test. To assess for overfitting of the data, we randomly assigned each case a “0” or “1” to split them into a training cohort (Group “0”) and a separate test cohort (Group “1”). For each cohort, we again determined the outcome dependence of SAC and variability as described above. An *R* ^2^ analysis was performed to assess correlation between median AF CL and outcomes.

## 3 RESULTS

### 3.1 Patient population

The study cohort consisted of data from 64 patients with persistent AF who underwent FIRM mapping and ablation at Erasmus Medical Center in Rotterdam, the Netherlands from March 4, 2015 through November 1, 2017. The mean age was 61.8 ± 8.9 years and 77% of patients were female, possibly owing to the selection of long‐standing persistent patients for the original FIRM mapping and ablation procedure. The mean AF duration was 4.9 ± 3.6 years and 46% of patients had undergone prior PVI. Baseline patient demographics are shown in Table 1.

**Table 1:** Demographic and main clinical data of the patient cohort

	Total	Recurrent AF by 12 months postablation	Free from AF at 12 months postablation
Number of patients, n	64	36	28
Age (years) (mean, *SD*)	61.8 ± 8.9	63.5 ± 7.4	59.6 ± 10.1
Sex (male), *n* (%)	49 (77%)	25 (69%)	24 (86%)
AF duration (years) (mean, *SD*)	4.9 ± 3.6	5.5 ± 3.9	4.3 ± 3.0
Prior pulmonary vein isolation, *n* (%)	30 (46%)	17 (47%)	13 (46%)
Hypertension, *n* (%)	39 (60%)	26 (72%)	13 (46%)
Hyperlipidemia, *n* (%)	17 (27%)	12 (33%)	5 (18%)
Diabetes mellitus, *n* (%)	12 (19%)	7 (19%)	5 (18%)
Sleep apnea, *n* (%)	3 (4.7%)	0 (0%)	3 (11%)
Ischemic heart disease, n (%)	14 (22%)	10 (29%)	4 (14%)
Dilated cardiomyopathy, n (%)	12 (19%)	0 (0%)	4 (14%)
Left ventricle ejection fraction (%) (mean, *SD*)	56 ± 10	57 ± 11	55 ± 8
Body mass index, *n* (%)	28.1 ± 4.5	28.5 ± 4.3	27.8 ± 4.7
Left atrial size (mm) (mean, *SD*)	45.7 ± 6.7	46.8 ± 6.8	44.3 ± 6.4
CHA_2_DS_2_‐VASc‐score (mean, *SD*)	1.83 ± 1.38	2.08 ± 1.3	1.5 ± 1.4

Abbreviations: AF, atrial fibrillation; CHA_2_DS_2_‐VASc‐score, risk stratification for stroke of AF patients.

At the time of the procedure, 14% of patients required AF induction before mapping. From these patients' procedures, 372 1‐min raw intracardiac unipolar electrogram recordings taken from 123 atria both pre‐ and post‐ablation using a 64‐pole basket catheter and were analyzed and post‐processed. Over half of patients (35/64, 54%) developed recurrent AF within 12 months post‐ablation. Procedural data are summarized in Table 2.

**Table 2:** Procedural data of the patient cohort

AF initiation required at the beginning of the procedure	9/64 (14%)
Termination during procedure, *n* (%)	24/64 (38%)
Recurrence within 12 months, *n* (%)	35/64 (54%)
Recurrence after termination, *n* (% of termination cases)	7/24 (29%)
Fluoroscopy time (min) (mean, *SD*)	32 ± 11
Procedure time (min) (mean, *SD*)	238 ± 68
Radiofrequency application duration (s) (mean, *SD*)	2438 ± 1228

Abbreviation: AF, atrial fibrillation.

### 3.2 Defining the activity threshold for clinically relevant sources

**Figure 2:**
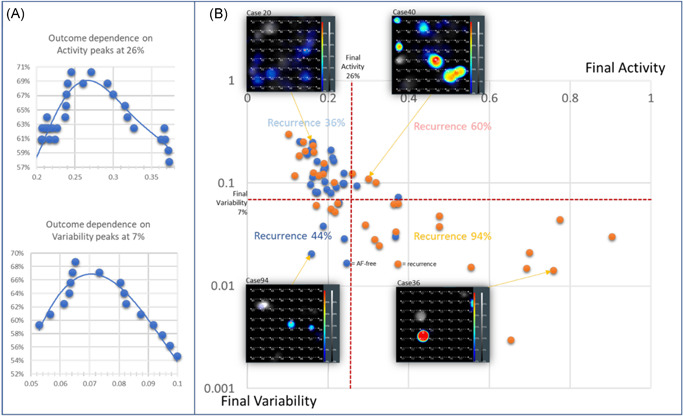
Electrographic flow (EGF) source activity and variability corelates with outcome in 64 persistent atrial fibrillation (AF) patients. (A) Accuracy of prediction of AF recurrence during 3‐, 6‐ or 12‐month follow‐up by both parameters was 69% and 67% and peaked at >26% activity and 7% variability, respectively. (B) Cases with both parameters below threshold showed 36% recurrence while cases with both parameters above threshold were almost exclusively recurrent (94%) with only one exception. One‐minute EGF source maps of four representative cases from all four quadrants are shown in the inlays. While cases below threshold show a more complex source pattern, cases above thresholds typically show single stable sources

The correlation of source parameters with procedural outcome revealed that outcome dependence on SAC level peaks at 26% (Figure 2). Selecting a statistically derived threshold for SAC of >26% allowed us to define a clinically relevant level of SAC. Though not statistically significant given the limited number of patients included in the present study, there did appear to be a second cut‐point for highly active sources with SAC > 35%. To assess how well these statistical analyses generalize to an independent data set, we randomized all 64 cases into either a development or a validation cohort and performed threshold detection in each unique group of patients separately. Among the 32 cases in the development cohort, the outcome dependence on SAC peaked at 27% with 70% accuracy and among the 32 cases in the validation cohort, the threshold was derived at 27.6% with 70% accuracy.

### 3.3 Change in median AF CL pre‐ and post‐ablation of sources

For all sources with SAC above threshold, the median AF CL pre‐ablation was 176 ± 31 ms and post‐ablation increased to 183 ± 35 ms. Thus, ablation increased the AF CL by 7 ± 14 ms. No correlation of AF CL or change in AF CL with outcome was observed. The increase in AF CL was weak, but not significantly correlated with the decrease of SAC by ablation, *R* ^2^ = 0.025.

### 3.4 EGF mapping‐based AF source signature types

By analyzing the correlation of source parameters with procedural outcome, we identified two distinct AF EGF signatures based on the activity levels of visualized sources on the EGF map. If at least one atrium showed a source with SAC > 26%, the patient's EGF signature was designated as S‐type. However, if there were no sources with SAC > 26%, the patient was felt not to have a relevant source above threshold and the patient's EGF signature was designated as C‐type. The highest EGF SAC pre‐ablation determined which atrium was chosen as the relevant one for post‐ablation analysis and outcomes correlation. Sources with high SAC in the RA septum that appeared to be the result of back‐propagation of excitation from the LA were excluded.

S‐type: Patients with individual stable sources with SAC above threshold, i.e., SAC of leading source >26% are classified as having an S‐Type EGF signature with source‐dependent AF because of the important contribution of the active sources above threshold to the initiation and maintenance of the patient's AF.

C‐type: Patients with no truly stable active source pattern and no leading source with SAC > 26% were characterized as having a C‐type EGF signature consistent with source‐independent AF because of the generalized chaotic electrical activity of the atrium in the absence of singular driving sources.

### 3.5 Correlation of EGF signature type and clinical outcomes

As shown in Figure 2A,B, final SAC and final variability of the leading source based on the EGF map generated from the final basket recording taken at the end of the ablation procedure correlated with outcome. Cases with AF recurrence at 3‐, 6‐, or 12‐month follow‐up showed a mean final SAC 34%; while those who remained AF‐free had leading sources with significantly lower mean final SAC 21% (*p* = .0006; *t*‐test, single‐sided, unpaired, different variances). Outcome dependence on final SAC and final variability was tested by plotting the percentage of correctly predicted outcomes with respect to a cut‐off value for these parameters (Figure 2A). Outcome dependence peaked at 26% for SAC and at 7% for variability with a best prediction accuracy of 69% and 67%, respectively (determined by the peak of a fifth order polynomial fit to the outcome accuracy vs. activity and variability, respectively). Patients with final SAC and variability above both thresholds had 94% recurrence, while recurrence was only 36% for patients with leading SAC and variability below threshold (Figure 2B, *χ* ^2^ *p* = .0001).

**Figure 3:**
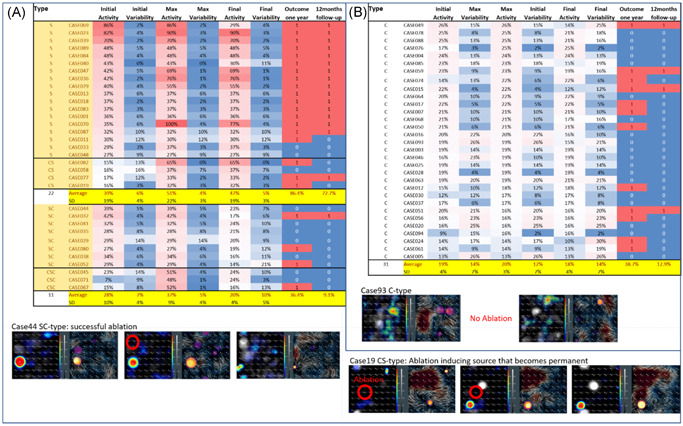
Patients classified into S‐ and C‐types and their initial, maximal, and final activities and variabilities of the leading sources versus outcome at any follow‐up (second‐to‐last column) or at 12‐month follow‐up (last column). Color code of activity and variability values ranges from 100% (red) over 20% (white) to 0% (blue). In the outcome columns, recurrence (1) is indicated in red and freedom from atrial fibrillation (AF) (0) is indicated in blue

By classifying patients according to their pre‐ablation EGF signature type, there were 26 patients (41%) with S‐type EGF signatures and 38 patients (59%) with C‐type EGF signatures. Summary statistics for each group are provided in Table 1. In total, 18 of these 26 active sources were focal while 8 were rotational. EGF maps generated from the post‐ablation recordings of the 26 S‐type patients showed that only 8 had their leading source successfully eliminated such that SAC decreased to ≤ 26%, the threshold above which defines a clinically relevant, active source (Figure 3A). By 12 months post‐ablation, 3 (38%) of the 8 with successful ablation showed a recurrence (a similar percentage as the 39% for those patients with a pre‐ablation C‐type EGF signature, Figure 3B). In contrast, 16 of the remaining 18 patients with S‐type EGF signatures (89%) who still had sources above threshold after ablation suffered AF recurrence. The two patients in this group that did not experience AF recurrence within 12 months post‐ablation were those who had the lowest initial SAC of 29% and 27% (Figure 3A).

In patients with a C‐type EGF signature with source‐independent AF, in whom leading SAC at baseline was already below the threshold to predict freedom from AF—FIRM‐guided ablation did not result in significant improvement in SAC measured on post‐hoc EGF maps (19 ± 4% initial vs. 18 ± 4% final activity) and the overall freedom from AF was 61%. For the 12 C‐type patients in whom FIRM‐guided ablation resulted in a reduction in SAC by the end of the procedure, the rate of freedom from AF at 1 year was 50%, suggesting that targeted ablation beyond PVI may not improve outcomes in patients with C‐type EGF signatures.

However, by analyzing and post‐processing the 64‐pole basket recordings taken throughout these cases, it was evident that while ablation in the correct location can eliminate sources, it can in some cases activate previously subthreshold sources. There were seven patients who started out with C‐type EGF signatures but were found to develop increased SAC in subsequent recordings obtained over the course of their ablation procedure. Thus, 33 of the 64 patients demonstrated a maximal SAC > 26% on any recording during the procedure and 23 of these patients (70%) experienced AF recurrence within 12 months postablation. On the contrary, among the 31 patients whose maximal SAC was ≤ 26% on any recording taken over the course of their procedure, only 12 patients (39%) experienced an AF recurrence within 12 months post‐ablation.

Accordingly, the presence or absence of SAC above threshold on EGF maps created from the final recordings at the conclusion of the ablation procedure also correlated with clinical outcomes. For the 22 patients in whom the EGF map generated from their final 64‐pole basket recording revealed an S‐type EGF signature with mean SAC 47%, 19 of them (86.4%) experienced AF recurrence by 1‐year post‐ablation. For the 42 patients in whom their final EGF map revealed a C‐type source signature with mean SAC 18.4%, only 16 of them (38.1%) experienced AF recurrence by 1‐year post‐ablation. Similarly, among the 39 patients who developed recurrent AF within 1 year of their procedure, their mean SAC on the final EGF map was 34.0%. Among the 29 patients who remained AF‐free at 1‐year post‐ablation, their mean SAC on the final EGF map was subthreshold at only 20.9%.

There were only 11 patients (17.1%) where FIRM mapping and ablation yielded a decrease in SAC between the EGF map with the highest value compared with the EGF map generated from the final post‐ablation recording. For these patients in whom FIRM‐guided ablation happened to eliminate the active source as determined by the post‐hoc EGF maps, 7 (63.6%) remained AF‐free at 1‐year post‐ablation compared with the 22 patients who ended the procedure with the final basket recording still showing an S‐type EGF signature on their post‐processed EGF maps and 86.4% developed recurrent AF within the year following the procedure (*χ* ^2^ *p* = .003).

## 4 DISCUSSION

In this study, the potential value of EGF mapping in patients undergoing catheter ablation for persistent AF was evaluated. The major finding of this study is that EGF mapping is capable of detecting active sources in the human atria during ongoing AF. Elimination of these active sources when their SAC is above threshold appears to be associated with improved clinical outcomes, while the presence of active sources with SAC above threshold at the conclusion of an ablation procedure correlates with a lower rate of freedom from AF at 1‐year post‐ablation. The ability to classify patients as having source‐dependent AF (S‐type EGF signature) versus source‐independent AF (C‐type EGF signature) may prove clinically useful for stratifying persistent and long‐standing persistent AF patients that will benefit from PVI alone and those that will require PVI in addition to targeted extra‐PV ablation.

### 4.1 Catheter ablation in patients with paroxysmal versus persistent AF

Circumferential isolation of the PVs has become a widely accepted and effective treatment strategy for patients with symptomatic drug‐refractory paroxysmal AF with favorable long‐term clinical success rates.^1^, ^2^, ^5^ Although PVI has been established as the cornerstone nonpharmacological AF treatment even for persistent AF patients, catheter ablation approaches for persistent AF remain challenging with limited long‐term success rates.^2^, ^6^, ^7^, ^18^ The optimal ablation strategy for persistent AF remains controversial and increasingly, the detection and elimination of AF sources within the atrial substrate is considered to play a key role.^5^, ^15^ To achieve a higher long‐term arrhythmia‐free survival after catheter ablation of persistent AF, additional ablation strategies have been utilized, most of them targeting non‐PV AF sources.^8^, ^19^, ^20^, ^21^, ^22^ Attempts to perform electrogram‐based quantitative approaches to guide ablation procedures targeting sites with maximal dominant frequency (DF) or complex fractionated atrial electrograms have been studied; however, the use of spectral analysis and AF CL analysis to localize high‐frequency sites relies on empirically derived definitions and the clinical application of these techniques remains limited due to its time‐consuming nature, lack of reproducibility, and inconsistent results.^23^, ^24^, ^25^, ^26^, ^27^ Because activation rate is a key parameter, CL measurements are preferable to determine DF; however, the spectrum of a signal is not solely determined by its CL, but also by signal morphology, changes in amplitude, and irregularity of intervals during AF.^28^ These techniques also do not account for underlying AF pathophysiologic mechanisms and while improved outcomes have been reported when maximal DF site ablation led to a significant DF reduction, this was achieved in the majority of paroxysmal AF patients with PVI only while prospective ablation of DF sites plus PVI in persistent AF patients resulted in low termination rates and failed to improve 1 year freedom from atrial arrhythmias over PVI alone.^26^, ^27^, ^29^ That a significant correlation was not seen between median AF CL and outcome in our retrospective study is not surprising given the fundamental differences in signal processing techniques employed between the EGF processing pipeline and a nominal signal analysis method.

So far, alternative technologies used for the identification of non‐PV AF sources are based on phase mapping or activation mapping.^8^, ^19^, ^20^, ^22^, ^30^, ^31^, ^32^ Whereas the initial results of FIRM‐guided AF ablation were promising, several studies reported inconsistent efficacy of AF driver ablation.^20^, ^33^, ^34^, ^35^, ^36^ These currently available technologies have some limitations, such as the limited spatiotemporal resolution and the inability to differentiate between active and passive rotational phenomenona.^8^, ^19^, ^20^, ^22^, ^30^, ^31^ The fact that FIRM‐mapping cannot discriminate passive flow disturbances caused by abnormal atrial substrate from active sources critical for initiating and maintaining AF may contribute to the conflicting published results of AF driver elimination during FIRM‐guided ablation procedures and the varying long‐term freedom from AF thereafter.^15^, ^16^, ^20^, ^33^, ^34^, ^35^, ^36^, ^37^ Additionally, the ablation of passive atrial structures leads to extended atrial scar formation, which may increase the occurrence of iatrogenic tachyarrhythmias and worsen overall long‐term ablation success rates.^15^, ^16^

### 4.2 Interaction of triggers, sources, and drivers in persistent AF

Most AF patients stochastically switch between AF and SR. Prodromal signs of AF are supraventricular extrasystoles while the switch to SR is sometimes preceded by an increased and/or regularized CL of AF before spontaneous termination.^38^, ^39^, ^40^ Triggers that induce AF originate in the PVs or alternatively represent focal sources of automaticity in the atrial wall myocardium.^4^, ^41^, ^42^ AF drivers, in contrast, are source activities that sustain fibrillatory electrical activity in the atrium and may include both re‐entrant sources as well as focal sources at any location within the myocardium.^43^, ^44^, ^45^ There is no necessary distinction between focal sources that trigger versus those that drive AF.^43^, ^44^

**Figure 4:**
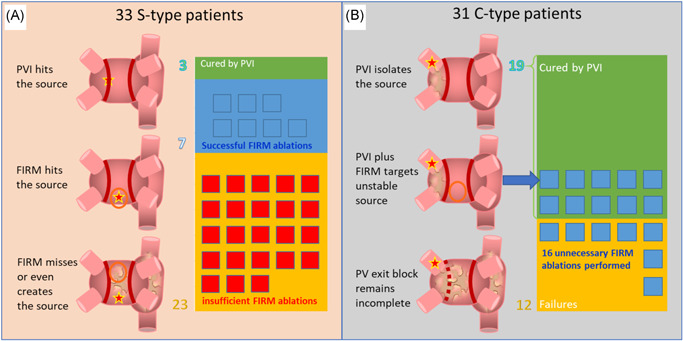
Summary of pulmonary vein (PV) and extra‐PV sources identified by electrographic flow (EGF) mapping and sorted by EGF source signature subtype and whether these EGF sources were ablated during pulmonary vein isolation (PVI) and/or focal impulse and rotor modulation‐guided ablation, sorted by outcome

Figure 4 depicts the various locations of AF sources and patient EGF signature classification in relation to their ablation procedures. Starting with the 64 patients analyzed in Figure 3, 33 patients had source‐dependent AF or an S‐type EGF signature. These patients were seen to have sources above threshold in the atria outside the PVs. In the three cases where the source was located on the PVI line, the patient remained AF‐free at 1‐year post‐PVI. In seven cases, the source identified by the post‐hoc EGF mapping had also been identified and successfully ablated during the original FIRM‐guided ablation procedure and these patients also remained AF‐free at 1‐year post‐ablation. However, in 24 cases, post‐hoc EGF maps identified sources that were either not seen during FIRM mapping or developed after FIRM‐guided ablation was performed.

For patients with source‐independent AF or a C‐type EGF signature, that is, no extra‐PV sources above threshold in the RA or LA; the PVs are the likely pathophysiologic mechanisms driving their AF. Of these 30 patients with C‐type EGF signatures, in 19 of them FIRM mapping identified a target for ablation in the atrial wall; however, on post‐hoc EGF mapping, there was no source above threshold identified. The 11 recurrences among these patients were potentially due to electrical reconnection of the PVs.

### 4.3 Study limitations

The primary limitation is that we are retrospectively analyzing data that were prospectively collected from a small cohort of patients who underwent FIRM mapping and ablation. Another limitation is that despite the fact that all patients were treated using a 3D electro‐anatomical mapping system, the basket position related to the patient's anatomy was not recorded in a standardized manner. Unless screenshots were taken using the CARTO (Biosense Webster) or the NavX (Abbott) systems during the procedure, the basket position was no longer retrievable and thus, it is not possible to ensure that basket recordings reflected complete coverage of the atria. As such, putative AF sources that would have been identified with EGF mapping may not have been detected because basket recordings did not adequately cover the atrial endocardial surface. In future prospective studies with intraprocedural EGF mapping, emphasis should be placed on the correlation of EGF‐identified sources and their anatomical locations. Similarly, because this study was post‐processing 1‐min raw unipolar intracardiac electrogram signals, assumptions were made including that all 64 pins of the basket catheter were properly pinned and no additional filter settings were applied to the recordings. Finally, regarding the threshold determination for clinically relevant SAC, because the outcomes of all cases were used to determine the SAC threshold of 26%, it is possible that this resulted in over‐fitting the results to find the threshold.

## 5 CONCLUSION

EGF mapping to identify EGF signature‐based AF subtypes may provide important prognostic information based on the characteristics of active leading sources visualized during persistent AF. Final SAC of the leading source as determined by 64‐pole basket catheter recordings taken at the end of the ablation procedure may correlate with the clinical outcome of an ablation procedure suggesting that only sources above a threshold of SAC > 26% represent relevant drivers/triggers of AF. Classification of persistent AF subtypes based on EGF signatures may help to differentiate those patients with source‐independent AF who would benefit from PVI‐alone (C‐type EGF signatures) versus those with source‐dependent AF requiring PVI plus targeted ablation of their active, clinically relevant extra‐PV sources (S‐type EGF signatures) to improve clinical outcomes.

## Data Availability

The data that support the findings of this study are available from the corresponding author upon reasonable request.
